# Clinicopathological and prognostic value of long noncoding RNA SNHG7 in cancers: a meta-analysis and bioinformatics

**DOI:** 10.18632/aging.203650

**Published:** 2021-10-29

**Authors:** June Wang, Shenlin Du, Chen Wang, Zinian Zhu, Baocheng Xie, Bashan Zhang

**Affiliations:** 1 Central Laboratory, Affiliated Dongguan Hospital, Southern Medical University, Dongguan, China; 2 Department of Pharmacy, Affiliated Dongguan Hospital, Southern Medical University, Dongguan, China; 3 Clinical Laboratory, Affiliated Dongguan Hospital, Southern Medical University, Dongguan, China; 4 Center for Gene Diagnosis, Zhongnan Hospital of Wuhan University, Wuhan, China

**Keywords:** lncRNA, SNHG7, cancer, clinical parameters, prognosis

## Abstract

The long intergenic non-coding RNA SNHG7 has been reported to be abnormally expressed in many types of cancer, the results remain controversial. In this study, a meta-analysis was performed to evaluate the clinicopathologic and prognostic value of SNHG7 in cancers. Electronic databases of PubMed, Web of Science, Cochrane Library and Embase were used to search relevant studies. A combined hazard ratio (HR) and its corresponding 95% confidence interval (CI) were used to assess the association between SNHG7 expression and prognosis in cancer patients. Pooled odds ratio (OR) and 95% CI were calculated to elaborate the association between SNHG7 expression and clinicopathological features in cancers. Besides, the data from The Cancer Genome Atlas (TCGA) dataset was used to validate the results. In total, eighteen studies compromising 1303 participants were enrolled in this analysis. The pooled results showed increased SNHG7 expression could predict unfavorable overall survival (OS) (HR = 1.75, 95%CI = 1.52–2.02, P = 0.000). Analysis stratified by follow-up time, cancer types, analysis types, sample sizes and cut off further verified the prognostic value of SNHG7. Additionally, elevated SNHG7 expression was correlated with TNM stage (OR: 3.31, 95%CI = 2.29–4.80, P = 0.000), lymph node metastasis (OR = 3.32, 95%CI = 1.61–6.83, P = 0.004), and tumor differentiation (OR = 1.92, 95%CI = 1.22–3.03, P =0.005) in patients with cancers. Excavation of TCGA dataset valuated that SNHG7 was upregulated in some cancers and predicted worse OS, which partially confirmed our results in this meta-analysis.

## INTRODUCTION

Cancer has been recognized as one of the most serious public health problems on a global scale. Due to changes in environmental factors and socio-economic development, the incidence and mortality of cancer are rising rapidly [1, 2]. It’s predicted that people affected by cancer will reach approximately 11.4 million by 2020, and one-third of the population will get cancer in their lifetime [3]. Despite enormous progress in the management and therapy of tumors over the past decade, the five-year survival rate for tumors is still not optimistic, mainly because many cancer patients have entered an advanced stage when they were diagnosed [4]. Therefore, it remains a high unmet need to discover new and effective biomarkers at an early stage to reduce cancer-related mortality.

Long non-coding RNAs (lncRNAs) which are a group of >200 nucleotides length RNA molecules do not contain functional open reading frame [5, 6]. Many lncRNAs defined as oncogenes and tumor suppressor genes in various types of cancer, effect on the cancer cell proliferation and apoptosis and involve in cancer invasion and metastasis [7–9]. For example, highly expressed lncHOTAIR could regulate cancer differentiation, distant metastasis in colorectal cancer tissues and closely related to TNM stage [10]. It has been reported that overexpressed LncSTCAT16 is a potent molecular target for inhibition of the cell proliferation and invasion in gastric cancer [11]. The up-regulation of LncPANDAR which promotes cell proliferation has been proved to related to poor prognosis of cervical cancer [12]. Besides, many researchers believed that some of lncRNAs could become potential therapeutic targets for monitoring cancer, predicting prognosis and evaluation of treatment effect [13, 14].

Small nucleolar RNA host gene 7 (SNHG7) has been regarded as a potential oncogene which is located on the long arm of chromosome 9 at band q34.3. It has been found that dysregulation of SNHG7 associated with growth and progress of different kinds of cancer, such as breast cancer, pancreatic cancer, hepatocellular carcinoma and cervical cancer [15–18]. Furthermore, a growing number of studies have explored the prognostic value of SNHG7 in various cancers, and suggested it can be exploited as a possible biomarker and therapeutic target for helping to improve diagnosis of cancer patients [19–23]. However, in current studies reporting the prognostic value of SNHG7 were subject to some qualifications, such as small sample size and contentious outcomes. Therefore, this quantitative meta-analysis cooperated with bioinformatics data was carried out to elucidate the prognostic value and clinicopathologic characteristics of SNHG7 in cancers.

## MATERIALS AND METHODS

### Literature searching strategies

For screening out all relevant studies up to August 2020, we conducted the online search of PubMed, Web of Science, Cochrane Library, and Embase. The keywords and MeSH terms used for the search were as follows: (“long non-coding RNA 7” or “SNHG7” or “small RNA host gene 7” or “lncSNHG7”) and (“neoplasm” OR “tumor” or “carcinoma” or “cancer”) and (“outcome” or “prognostic” or “prognosis”). Through searching other references cited by these retrieval studies, more related potential records were identified.

### Inclusion and exclusion criteria

The inclusion criteria for eligible studies were as follows: (a) measuring the expression of SNHG7; (b) valuable data that could reflect the relationship between SNHG7 level and clinicopathological features or prognosis; (c) sufficient data for obtaining the values of HRs and CIs to judge the survival outcomes. The exclusion criteria were as follows: (a) repetitive research; (b) studies unrelated to cancer; (c) non-human studies; (d) unable to get the HRs and 95%CI from raw data; (e) case reports, comment, and reviews.

### Quality evaluation and data extraction

Two reviewers (JEW and CW) extracted the data independently from all identified records. The article general information, tumor type, sample type, the number of involved patients, detection method, follow-up time, cut off value, outcome measure, hazard rate and SNHG7 evaluation score were extracted from each study. Some articles directly provided at least one of three values of OS, DFS and PFS, while we had to use Engauge Digitizer version 4.1 to obtain the above data from the rest of articles according to the method described by Tierney [23]. All of the selected studies with the Newcastle-Ottawa Scale (NOS) score of 7 or above were considered high-quality articles [24].

### Bioinformatics validation

We exploited online analysis software the Gene Expression Profiling Interactive Analysis (GEPIA) to further explore the differential expression of SNHG7 among cancer tissues. All data are based on The Cancer Genome Atlas (TCGA) database. and We set P < 0.01 as the cut-off value to compare TCGA various neoplasms data with normal group. In order to assess the correlation between SNHG7 expression and OS, we’ve chosen Kaplan-Meier method and log-rank test to calculate the survival analysis.

### Statistical analysis

All statistical analyses were completed by the STATA software version 15.0. The chi-squared Q test and the *I*^2^ statistic were used to observe the heterogeneity among studies. If the significant heterogeneity existed (*P* < 0.05 for Chi-squared test or *I*^2^ > 50%), random-effect model was used. If not, we used the fixed-effect model. Evaluation methods of the following correlation analyze, SNHG7 expression and prognosis in cancers: pooled HRs and 95% CIs; SNHG7 expression and clinicopathological features: combined odds ratio (OR) and 95% CIs; publication bias: funnel plots and Begg’s test. By removing individual study in sequence, we used sensitivity analysis to verify that the results were credible and stable. Furthermore, we defined the *p* value less than 0.05 as statistically significant.

## RESULTS

### Description of studies

**Figure 1:**
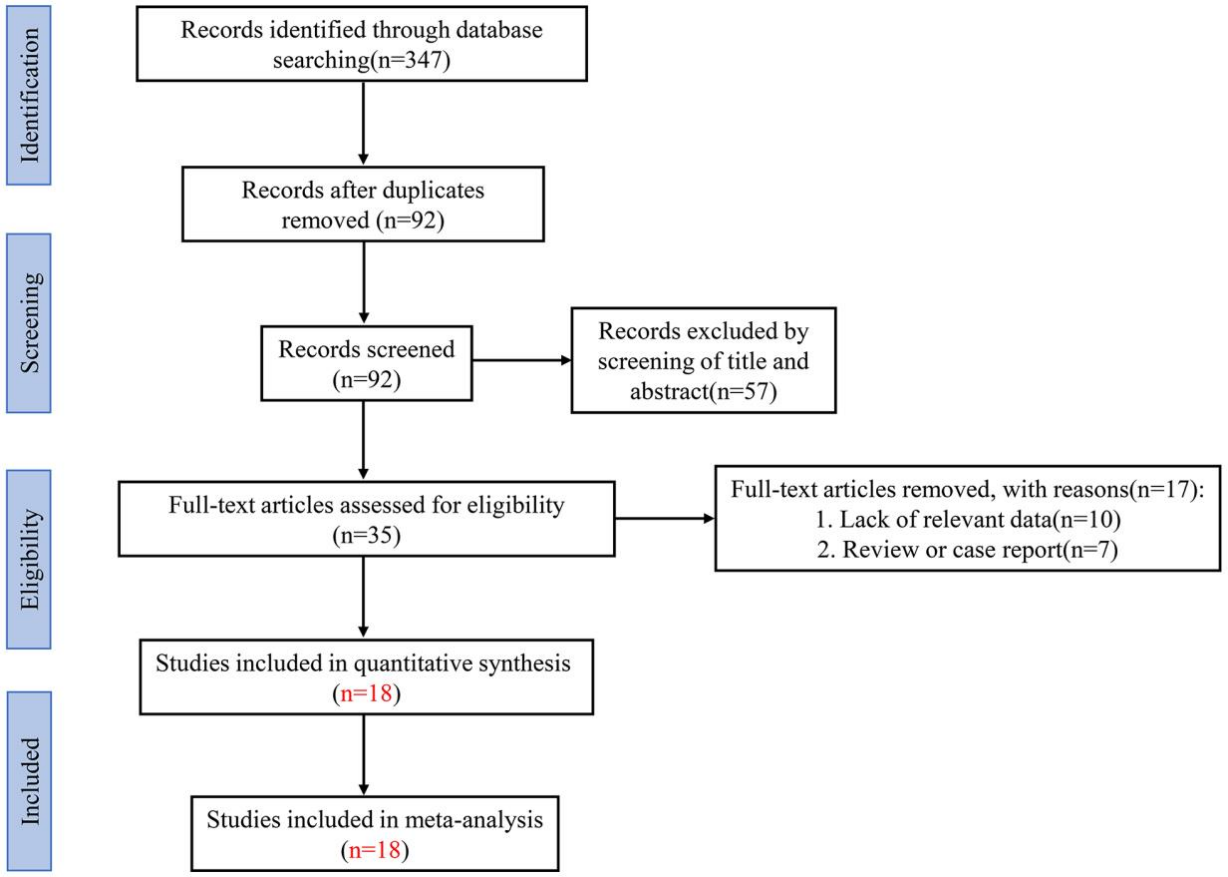
Flow diagram of the literature retrieval and selection in this meta-analysis.

The detailed steps and processes of the article retrieval and selection were displayed in Figure 1. A total of 347 articles were revealed in the initial literature search. However, we selected 35 studies in strict accordance with inclusion and exclusion criteria. Subsequently, after screening the full texts of the remaining 35 articles, another 17 studies were excluded. Finally, only 18 articles included 1303 cancer patients were fully in conformity with the screening criteria and enrolled in the current meta-analysis [15–18, 22, 23, 25–36].

The selected studies were all conducted in China, and their main characteristics have been summarized in Table 1. Publication years ranged from 2018 to 2020, and the sample sizes were between 30 and 162. These eligible researches contained 1303 patients, involved 11 types of cancers. There were three hepatocellular carcinoma studies, three colorectal cancer studies, two prostate cancer studies, two neuroblastoma studies, two cervical cancer studies, one bladder cancer study, one gastric cancer study, one pancreatic cancer study, one osteosarcoma study, one hypopharyngeal cancer study, and one breast cancer study. In these studies, SNHG7 expression levels were detected in tumors using the qRT-PCR method. The enrolled patients were divided into two groups according to the expression of SNHG7: high expression and low expression groups. All studies examined the association of SNHG7 with OS, and one study reported PFS and DFS respectively. Five studies have directly given HR values, and other thirteen studies only provided the Kaplan-Meier survival curves for calculating the HR values indirectly. Moreover, the number of studies involved in distance metastasis (DM), lymph node metastasis (LNM), TNM stage, and tumor differentiation was 6, 10, 6, and 6 respectively. All publications met the good quality in the NOS scoring system.

**Table 1:** Characteristics of studies in this meta-analysis.

**Study**	**Year**	**Country**	**Tumor types**	**Sample size**	**Sample**	**Detection method**	**Cut off value**	**Follow-up (year)**	**Outcome measures**	**Extract method**	**NOS**
Jia	2020	China	neuroblastoma	45	tissues	qRT-PCR	mean	5 years	OS	K-M Curve	8
Shen	2020	China	hepatocellular carcinoma	100	tissues	qRT-PCR	mean	5 years	PFS OS	K-M Curve	7
Zhang	2020	China	synchronous colorectal liver metastasis	96	serum	qRT-PCR	median	3 years	OS	Reported	8
Wu	2020	China	cervical cancer	51	tissues	qRT-PCR	median	5 years	OS	K-M Curve	7
Xia	2020	China	prostate cancer	127	tissues	qRT-PCR	mean	5 years	OS	Reported	8
Zhang	2020	China	gastric cancer	162	tissues	qRT-PCR	median	8 years	OS	Reported	9
Zeng	2019	China	cervical cancer	60	tissues	qRT-PCR	NA	5 years	OS	Reported	8
Yang	2019	China	hepatocellular carcinoma	80	tissues	qRT-PCR	median	5 years	OS	K-M Curve	8
Yao	2019	China	hepatic carcinoma	40	tissues	qRT-PCR	median	3 years	OS	K-M Curve	7
Cheng	2019	China	pancreatic cancer	40	tissues	qRT-PCR	NA	3.5 year	OS	K-M Curve	7
Wu	2019	China	hypopharyngeal cancer	73	tissues	qRT-PCR	median	5 years	OS	K-M Curve	8
Chi	2019	China	neuroblastoma	92	tissues	qRT-PCR	mean	5 years	OS	K-M Curve	8
Luo	2018	China	breast cancer	72	tissues	qRT-PCR	NA	6.5 years	OS	K-M Curve	7
Chen	2019	China	bladder cancer	92	tissues	qRT-PCR	NA	5 years	OS	K-M Curve	8
Deng	2018	China	osteosarcoma	30	tissues	qRT-PCR	median	5 years	OS	K-M Curve	7
Li	2018	China	colorectal cancer	53	tissues	qRT-PCR	NA	5 years	OS DFS	Reported/ K-M Curve	8
Qi	2018	China	prostate cancer	42	tissues	qRT-PCR	NA	5 years	OS	K-M Curve	7
Shan	2018	China	colorectal cancer	48	tissues	qRT-PCR	median	5.5 years	OS	K-M Curve	7

### The SNHG7 expression and disease prognosis

**Figure 2:**
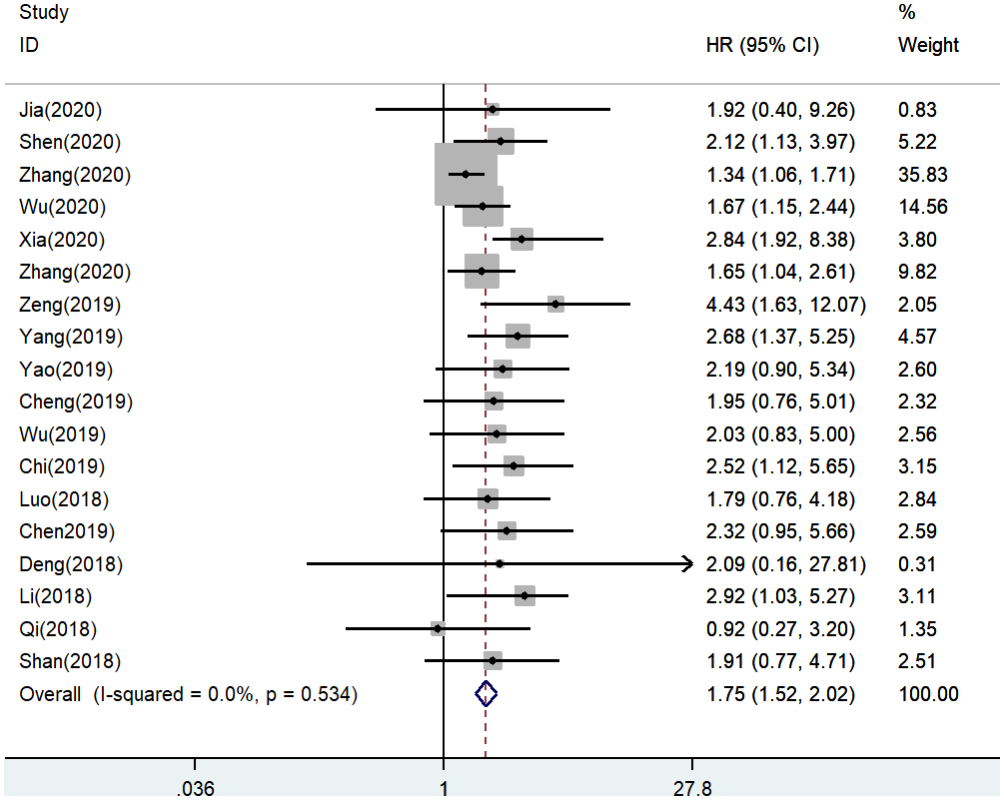
Forest plot for the association between SNHG7 expression with overall survival (OS).

A total of 18 eligible studies with a sample of 1303 patients were recruited to evaluate the expression level of SNHG7 on OS. The pooled HR was 1.75 (95% CI: 1.52-2.02, *P* = 0.000), which indicated that the high expression of SNHG7 was significantly related to shorter overall survival (Figure 2). Since there was no heterogeneity between the studies (*I*^2^ = 0.0%, *P* = 0.534), a fix-effect model was applied to analyze the HR and 95% CI of OS. Our analysis showed that the up-regulated SNHG7 expression had a positive correlation with the worse overall survival in cancer patients.

In order to further explore the correlation between SNHG7 expression and the OS, all included patients were classified, based on follow-up time, cancer types, analysis types, sample sizes and cut off, to perform the subgroup analysis of OS.

**Figure 3:**
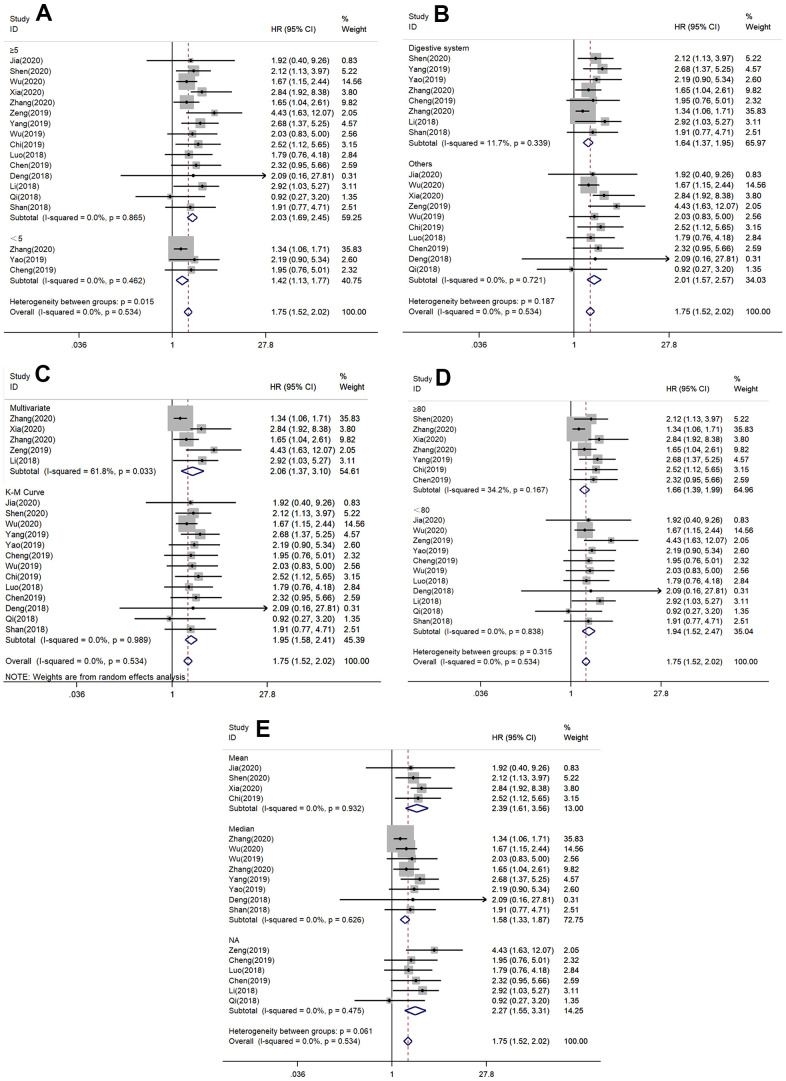
Forest plots of hazard ratios (HRs) for the relationship between SNHG7 expression and overall survival (OS): (**A**) stratified by follow-up time; (**B**) stratified by cancer type; (**C**) stratified by analysis type; (**D**) stratified by sample size; (**E**) stratified by cut off value.

As showed in Table 2, a strong correlation was discovered between increased expression of SNHG7 and poor OS in digestive system cancers (HR = 1.64, 95% CI =1.37-1.95, *P* < 0.000), other cancers (HR = 2.01, 95% CI = 1.57-2.57, *P* = 0.000). Simultaneously, the combined HRs of elevated SNHG7 expression on OS in patients with multivariate, K-M Curve respectively were 2.06 (95%=1.37-3.10, *P* = 0.001) and 1.95 (95% CI = 1.58-2.41, *P* = 0.000). Furthermore, in terms of follow-up time, sample sizes and cut off similar results, we observed that cancer patients with worse OS carried high expression of SNHG7 (Figure 3). The above results supported that SNHG7 could be considered as a prognostic indicator, for interfering with OS in cancer patients.

**Table 2:** Subgroup analysis of SNHG7 expression and overall survival (OS) in cancer patients.

**Subgroup analysis**	**No. of studies**	**No. of patients**	**Pooled HR(95%CI)**	***P***	**Heterogeneity**		**Model**
					***I^2^*(%)**	***P*-value**	
OS	18	1303	1.75(1.52-2.02)	0.000	0	0.534	Fixed
**Follow-up time**							
≥5 years	15	1127	2.03(1.69-2.45)	0.000	0	0.865	Fixed
<5years	3	176	1.42(1.13-1.77)	0.002	0	0.462	Fixed
**Tumor type**							
Digestive system cancer	8	619	1.64(1.37-1.95)	0.000	11.7	0.339	Fixed
Others	10	684	2.01(1.57-2.57)	0.000	0	0.721	Fixed
**HR estimation method**							
Multivariate	5	498	2.06(1.37-3.10)	0.001	61.8	0.033	Random
K-M Curve	13	805	1.95(1.58-2.41)	0.000	0	0.989	Fixed
**Number of patients**							
≥80	7	749	1.66(1.39-1.99)	0.000	34.2	0.167	Fixed
<80	11	554	1.94(1.52-2.47)	0.000	0	0.838	Fixed
**Cut-off**							
Mean	4	364	2.39(1.61-3.56)	0.000	0	0.932	Fixed
Median	8	580	1.58(1.33-1.87)	0.000	0	0.626	Fixed
NA	6	359	2.27(1.55-3.31)	0.000	0	0.475	Fixed

However, only two studies provided data for PFS or DFS, which made it impossible for us to perform a meta-analysis to pool the results. In the study of Li et al. [23] , the HR was 4.505, 95% CI was 1.98–10.309 (*P* = 0.0043). In the study of Shen et al. 17], HR was 1.73, 95% CI was 1.03–2.90 (*P* < 0.001). These two studies demonstrated that high SNHG7 expression was significantly related to poor DFS or shorter PFS.

### The SNHG7 expression and clinicopathological characteristics

**Figure 4:**
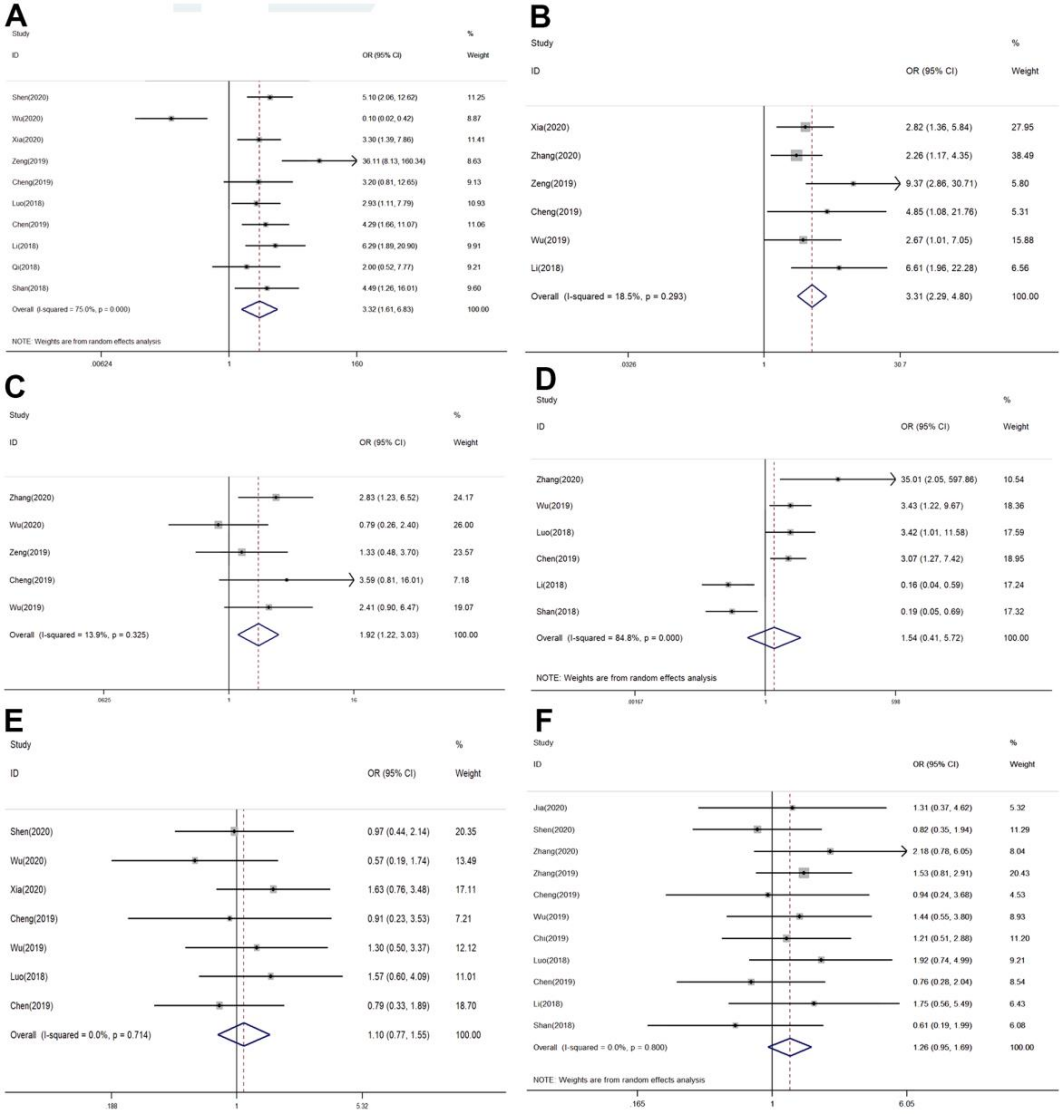
Forest plots of odds ratios (ORs) for the relationship between SNHG7 expression and clinical parameters: (**A**) lymph node metastasis; (**B**) TNM stage; (**C**) Tumor differentiation; (**D**) distant metastasis; (**E**) age; (**F**) gender.

A further meta-analysis of studies describing tumor characteristics was performed to determine whether the SNHG7 expression was related to the clinical- pathological parameters, including age, gender, TNM stage, lymph node metastases, distant metastasis, and tumor differentiation. The significant association between SNGH7 and lymph node metastases was proved by ten studies ten including 685 patients (positive vs. negative, OR = 3.32, 95% CI = 1.61–6.83, *P* = 0.001, Table 3 and Figure 4A). To solve the high heterogeneity, we chose the random-effects model (*I*^2^ = 75%, *P* = 0.000). Meanwhile, six studies involving 515 patients were included to evaluate the connection between SNHG7 and the TNM stage. There was no significantly heterogeneity difference in the studies (*I*^2^=18.5%, *P* = 0.293). The pooled OR (the value was 3.31, 95% CI: 2.29–4.80, *P* = 0.000) indicated that high expression level of SNHG7 was associated with advanced clinical stage. (Figure 4B).

**Table 3:** The correlation between SNHG7 expression and clinicopathological features.

**Clinicopathological features**	**No. of studies**	**No. of patients**	**OR (95% CI)**	***P***	**Heterogeneity**		**Model**
					***I^2^***	***P*-value**	
Age (≥60 vs. <60)	7	555	1.10 (0.77–1.55)	0.604	0.0%	0.714	Fixed
Gender (Female vs. male)	11	873	1.26 (0.95–1.69)	0.113	0.0%	0.800	Fixed
TNM stage (III–IV vs. I–II)	6	515	3.31 (2.29–4.80)	0.000	18.5%	0.293	Fixed
Lymph node metastases (Positive vs. negative)	10	685	3.32 (1.61–6.83)	0.001	75%	0.000	Random
Distant metastasis (Positive vs. negative)	6	478	1.54 (0.41–5.72)	0.522	84.8%	0.000	Random
Tumor differentiation (Poor vs. Moderate/Well)	5	320	1.92 (1.22–3.03)	0.005	13.9%	0.325	Fixed

There were five studies describing how SNGH7 involved in histological grading. The analysis showed that OR was 1.92, 95% CI was 1.22–3.03 (*P* =0.005, Figure 4C). These data supported poor histological grade was accompanied with elevated SNGH7 expression. A fixed-effects model was used for no heterogeneity in this analysis (*I*^2^ = 13.9%; *P* = 0.325). There was no statistical significance observed in DM (OR =1.54, 95% CI = 0.41–5.72, *P*=0.522), age (≥60 vs.< 60, OR = 1.10, 95% CI: 0.77-1.55, *P* = 0.604), and gender (female vs. male, OR = 1.26, 95% CI: 0.95-1.69, *P* = 0.113) (Figure 4D–4F).

### Sensitivity analysis and publication bias

**Figure 5:**
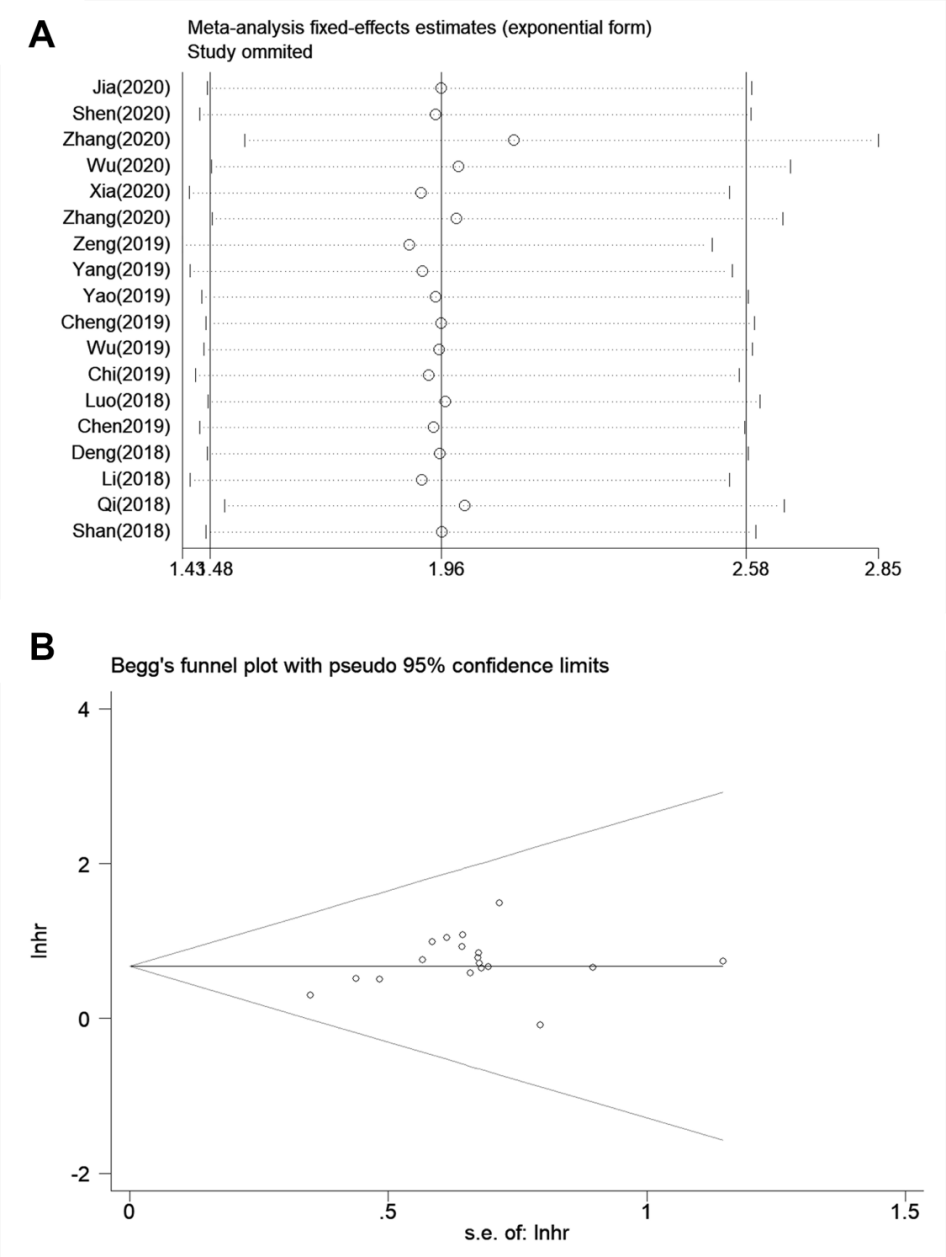
**Sensitivity analysis and publication bias for meta-analysis of SNHG7 and OS.** (**A**) Sensitivity analysis for meta-analysis of SNHG7 and OS. (**B**) Funnel plot of the publication bias for OS.

Sensitivity analysis was to examine whether the OS was interfered by individual studies. There was no significant influence on the pooled results when any individual study was removed. At last, we found that the HR values and the synthesized results were stable and reliable (Figure 5A). According to Begg’s tests, the symmetrical shape of the funnel plot indicated that no significant publication bias existed in the study (*P*=0.649, Figure 5B).

### Validation of TCGA dataset results

**Figure 6:**
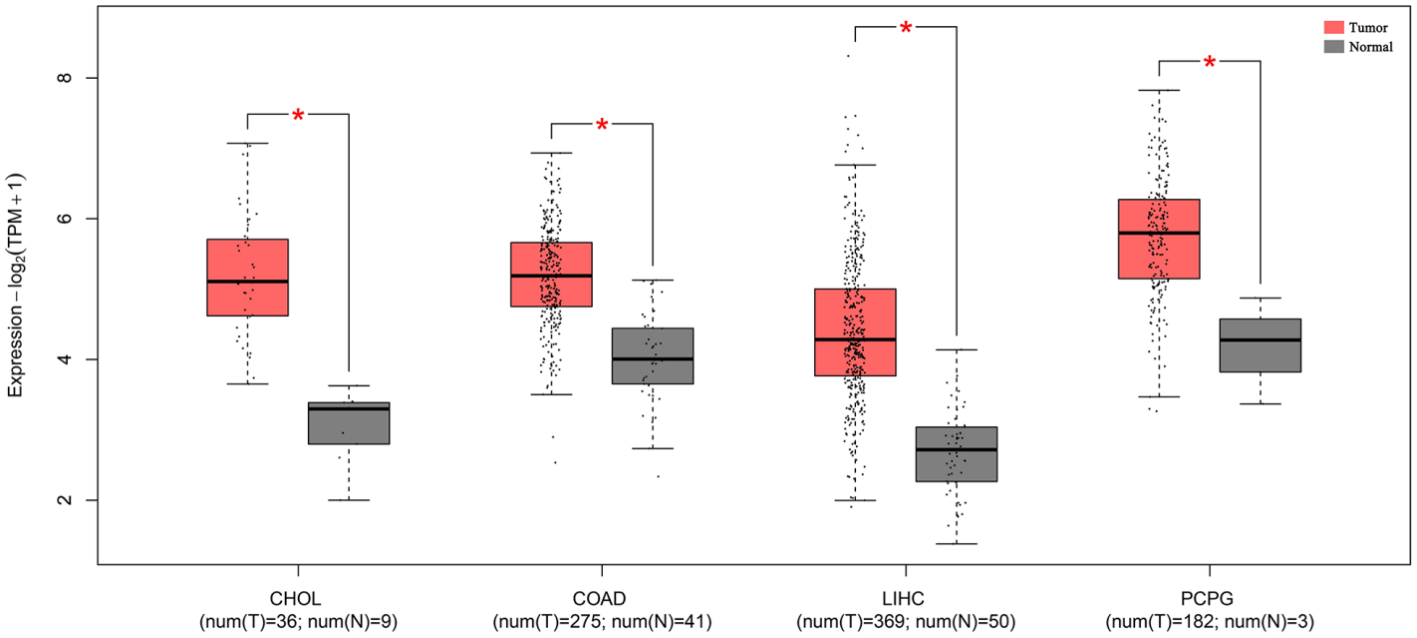
**The expression levels of SNHG7 in four kinds of cancer tissues and normal tissues.** “*”|Log2FC|>1 and P<0.01. Abbreviations: CHOL, cholangiocarcinoma; COAD, colon adenocarcinoma; LIHC, liver hepatocellular carcinoma; PCPG, pheochromocytoma and paraganglioma.

**Figure 7:**
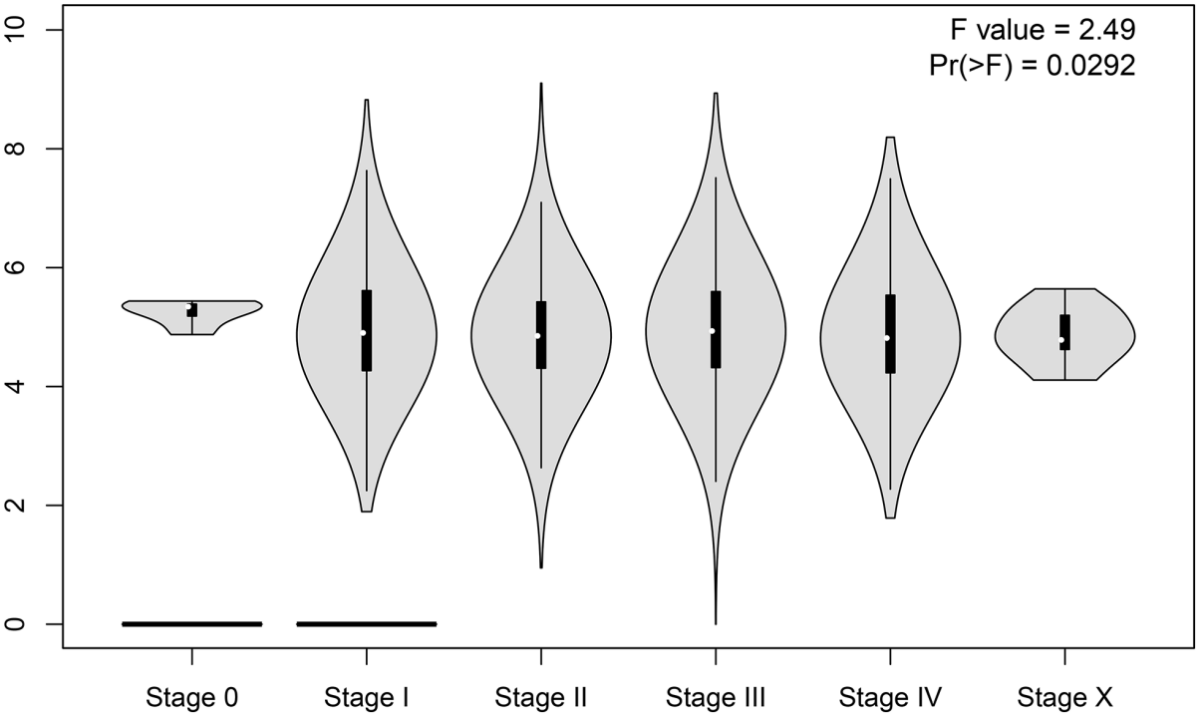
Violin plot of clinical stage of SNHG7 expression in human pancancers.

**Figure 8:**
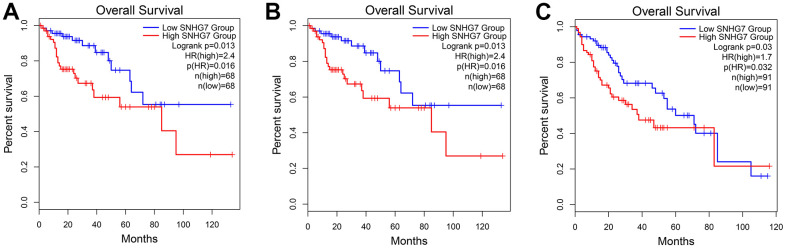
**Validation of the prognostic value of SNHG7 based on the TCGA database.** (**A**) The survival curve of patients with COAD; (**B**) The survival curve of patients with BLCA; (**C**) the survival curve of patients with LIHC. Abbreviations: COAD, colon adenocarcinoma; BLCA, bladder urothelial carcinoma; LIHC, liver hepatocellular carcinoma; TCGA, The Cancer Genome Atlas.

Using the expression data of SNHG7 from the TCGA dataset was to further validate its prognostic and clinical value in cancers. As shown in Figure 6, SNHG7 overexpression was identified in cholangiocarcinoma (CHOL), colon adenocarcinoma (COAD), liver hepatocellular carcinoma (LIHC), pheochromocytoma and paraganglioma (PCPG) (|Log2fold change (FC)| cutoff >1 and *P*<0.01). Moreover, the violin plot showed that SNHG7 expression was also significantly associated with the clinical stage of human cancers (*P* < 0.05, Figure 7). For clarifying the relationship between SNHG7 expression and prognosis, we used the GEPIA database to plot survival curve according to quartile grouping cut-off. It has revealed that SNHG7 upregulation was correlated with worse OS in COAD, bladder urothelial carcinoma (BLCA), LIHC (log-rank *P*<0.05, Figure 8), which partially confirmed our results in this meta-analysis.

## DISCUSSION

LncRNAs, a biomolecule with regard to tumor epigenetics, post transcriptional regulation, genome stability and ceRNA regulation, has increasingly been emphasized by oncology researchers [37, 38]. LncRNA can act as an oncogene or tumor suppressor gene, and directly or indirectly regulate cancer-related signal pathways to affect tumor development. For example, Liu et al. proved that lncRNA GASL1 inhibited cell proliferation and metastasis by sponging microRNA-106a in gastric carcinoma [39]. Xiang et al. confirmed that SNHG7, as an endogenous ceRNA, could regulate HK2 expression via sponging miR-143-3p to promote the proliferation of bladder cancer [40]. Besides, in hepatocellular carcinoma, lncRNA NKILA has been reported for suppressing NF-κB/Slug pathway mediated with epithelial-mesenchymal transition to inhibit tumor metastasis [41].

SNHG7 was considered as a novel oncogenic lncRNA for its abnormal expression in various types of cancer. A growing number of articles have shown how SNHG7 participated in running processes of molecular biological pathways in the occurrence and development of cancer. Li et al. [23]discovered that the expression of SNHG7 was dramatically up-regulated in hepatocellular carcinoma (HCC), while knockdown of SNHG7 expression could suppress the growth and metastasis of HCC cells [30]. In breast cancer (BC), some researchers suggested that the increased expression of SNHG7 could promote tumorigenesis and progression by EMT initiation and the activation of the Notch-1 pathway through interacting with miR-34a [42]. In addition, it has been reported that SNHG7 may conduct as an oncogene in colorectal cancer (CRC) progression, and increased expression of SNHG7 is related to cell proliferation, inhibition of cell apoptosis, and enhancement of liver metastasis of CRC [36]. It has proved that SNHG7 was able to regulate osteosarcoma (OS) cell vitality, migration, and invasion by activating sponging miR-34a, which targeted and suppressed the epithelial-mesenchymal transition (EMT) through the TGF-β/SMAD4 signaling pathway [35]. Through down-regulating expression of SNHG7, Wang et al. found that the proliferation of thyroid cancer (TC) cells could be inhibited and cell apoptosis was induced. Moreover, they indicated that the high expression of SNHG7 was positively correlated with increased tumor size and advanced TNM stage [43]. The study by Chen et al. revealed that the knockdown of SNHG7 expression promoted bladder cancer (BC) cell survival and proliferation via activating the ERK/MAPK signaling pathway [33]. In summary, we made conclusion that SNHG7 plays inconsistent roles in different cancer. Thus, a comprehensive analysis was performed to evaluate whether SNHG7 has the value of predicting the prognosis of tumor patients.

In our finding, lncRNA SNHG7 might be considered as an unsatisfactory prognosis factor for cancer patients. It is inferred that higher expression of SNHG7 would lead to poorer OS, the HR and 95% CI were 1.75 and 1.52–2.02 respectively. The subgroup meta-analysis stratified by follow-up time, cancer types, analysis types, sample sizes and cut off, also drew the above similar conclusions. Notably, the results from TCGA databases also indicated that in colorectal cancer, bladder cancer and hepatocellular carcinoma, the OS of patients with high SNHG7 expression was shorter than that of those with low SNHG7 expression, which partially identified and strengthened our results in this meta-analysis. Moreover, we explored the relationship between SNHG7 expression and clinical parameters. It was noticed that the exiting data supported a significant positive correlation between higher SNHG7 expression level and more advanced TNM, earlier lymph node metastasis, and poorer histological grade. Collectively, we believed that lncRNA SNHG7 was involved in the tumor development and progression, and may become a promising biomarker for prognosis in cancer patients.

Nevertheless, this study has some limitations that should be refined. First of all, due to the relatively small number of included patients, the study might be the lack of certain stringency. Second, the cut-off values including median, mean, and others for positive SNHG7 expression were not consistent in different studies, and lacking a uniform criterion. Third, all patients included in the studies were of Chinese descent. Fourth, the accuracy of HRs and 95% CIs extracted from Kaplan-Meier curves were not enough, it might be less than the data directly given in original articles. It might add the potential bias. Hence, the results should be interpreted cautiously.

## CONCLUSIONS

In summary, the higher lncRNA SNHG7 expression would lead to more advanced TNM, earlier lymph node metastasis, and poor histological grade. Thus, cancer patients with poorer OS were often accompanied with the increasing expression of lncRNA SNHG7. Our meta-analysis suggested that lncRNA SNHG7 could serve as a promising biomarker for prognosis with cancers. Still, all in all, we hope that some multi-centre, larger and higher-quality studies will be carried out to strengthen our results in the future.
